# Supplementary figures and images for: bra-miR167a Targets ARF8 and Negatively Regulates Arabidopsis thaliana Immunity against Plasmodiophora brassicae
Source: Int J Mol Sci. 2023 Jul 24;24(14):11850. doi: 10.3390/ijms241411850 (PMC10380745; doi:10.3390/ijms241411850)

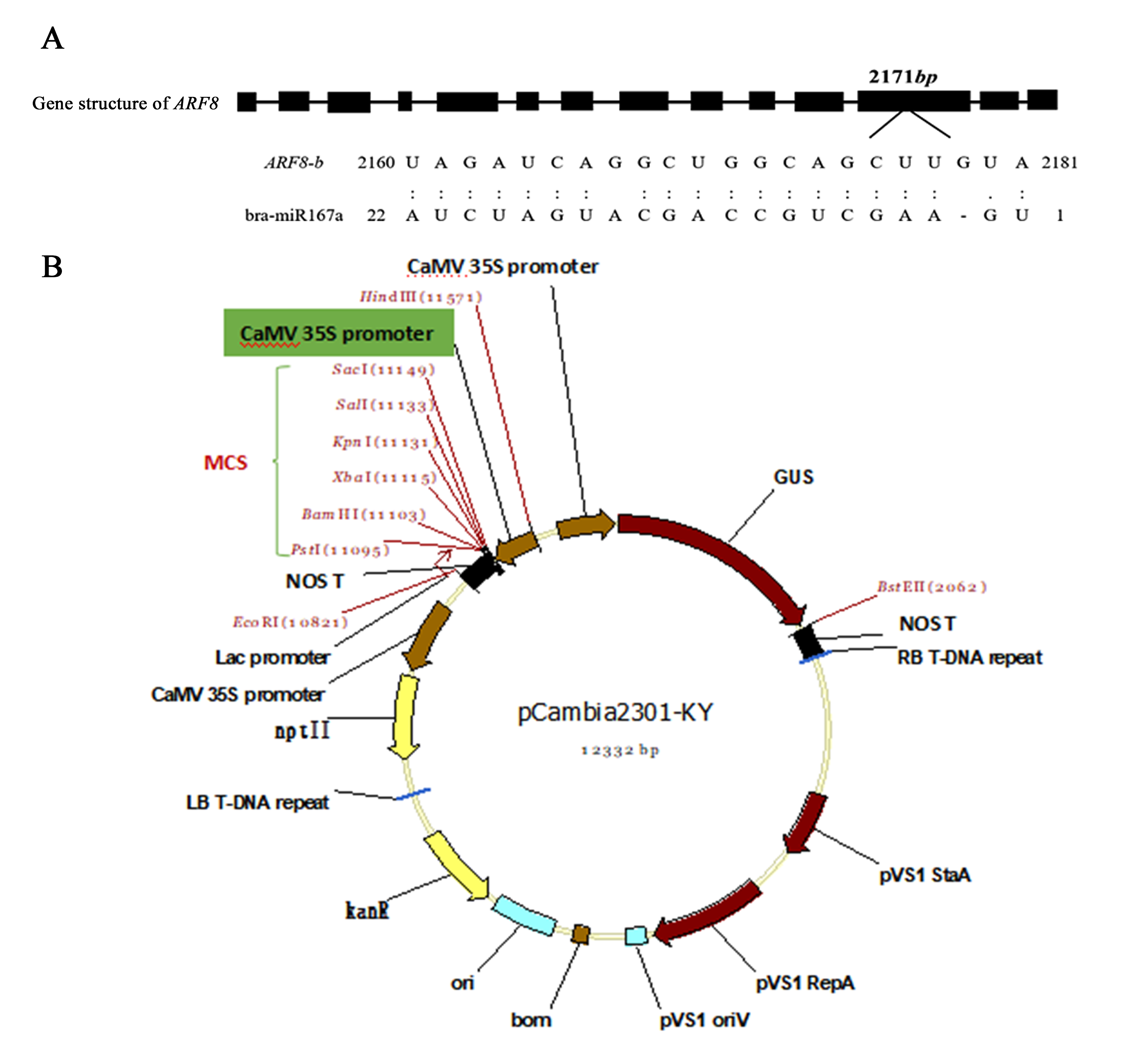

Supplement: Supplementary file 1 [file ijms-24-11850-s001.zip › supplmentary materials/Supplementary Figure 1.tif]
